# College and Hospital Notes

**Published:** 1900-12

**Authors:** 


### COLLEGE AND HOSPITAL NOTES.

The Buffalo General Hospital has adopted plans for an addition and were filed recently in the Bureau of Buildings. The estimated cost is $35,000. The additional building will be 38 feet wide and 96

feet long. It will be an extension of the present wing and will correspond in design to the new wing recently built. The same kind of materials, yellow brick and terra cotta, will be used. The addition will front on Elm Street. Architect George Cary prepared the plans and to Jared H. Tilden has been awarded the contract for building.

---

A NEW Emergency Hospital will be built on the corner of Eagle and Pine Streets, for the construction of which a contract has been awarded to John Lannen. Green and Wicks are the architects for the new building and the plans call for a three-story brick building with basement. The basement will be used for clinical purposes, the first floor will contain the primary wards and the second floor the secondary wards. The surgeons and attendants will be housed on the third floor. The Emergency Hospital is the accident branch of the Buffalo Hospital of the Sisters of Charity.

---

AT THE New York Skin and Cancer Hospital, Second Avenue, cor. 19th Street, the board of governors announces that Dr. L. Duncan Bulkley is giving a series of clinical lectures on diseases of the skin in the out-patient hall of the hospital, on Wednesday afternoons, that commenced November 7, 1900, at 4.15 o'clock. The course is free to the medical profession.

---

THE College of Physicians and Surgeons, of Chicago, has received a gift of $50,000 from two prominent physicians of that city. Dr. William E. Quine, dean of the school, gives $25,000 to endow the college library, and Dr. D. A. K. Steele gives $25,000 to endow the pathological laboratory.

These princely gifts are conditional upon the purchase of the trustees of the University of Illinois, of which the College of Physicians and Surgeons is a part, of the old west division high school building. All the negotiations for this purchase have been completed, however, and the transaction wants only the approval of the city council for its consummation. When the deeds have been signed the university trustees will fit up rooms for the library and the laboratory in the building, and the two physicians will at once turn over to them the endowment securities.

